# Analysis of Performance Indicators in Decision-Making and Execution in U14 Female Basketball Players

**DOI:** 10.3390/sports14090366

**Published:** 2026-08-24

**Authors:** Jorge Iglesias-Martín, Salvador Pérez-Muñoz, Alberto Rodríguez-Cayetano

**Affiliations:** 1 Faculty of Education, Pontifical University of Salamanca, 37007 Salamanca, Spain; jiglesiasma@upsa.es (J.I.-M.); sperezmu@upsa.es (S.P.-M.); 2 Research Group EGIIOFYD, Pontifical University of Salamanca, 37007 Salamanca, Spain

**Keywords:** female, offensive game performance, offensive game involvement, game positions, team sports, observational analysis

## Abstract

Female youth basketball remains an underexplored area of performance analysis. This study aimed to analyze the participation and offensive performance of players on a female youth basketball team across a season, assessing decision-making and execution in offensive plays according to playing position and contextual variables. An observational methodology was applied using the Game Performance Assessment Instrument (GPAI) across 15 matches involving a youth team (*n* = 11; mean age 13.89 ± 0.25 years). A total of 9450 offensive acts (dribbles, passes, shots, screens, and off-ball movements) were analyzed, calculating participation, execution, decision-making, and game performance metrics by playing position (guards, forwards, and centers) and four contextual variables. A multivariate analysis of variance (MANOVA) was applied, and univariate analyses of variance (ANOVA) were subsequently conducted to identify the specific index in which differences were located. Results showed the highest values across all indices for guards (Participation = 45.12%; Execution = 0.85; Decision-Making = 0.92; Game Performance = 0.89) and the lowest for centers (26.28%, 0.77, 0.85, 0.81, respectively), confirming differentiated offensive profiles. Regarding contextual variables, playing at home improved offensive performance for all positions, while performance declined in all positions as proximity to the opponent’s basket increased. By contrast, the game period showed position-specific differences, and also previous wins modulated participation and offensive performance, reflecting the influence of perceived opponent quality, with effects varying by position. These findings highlight the need for position- and context-specific training to enhance offensive performance for this team’s players.

## 1. Introduction

Women’s basketball has steadily experienced growth over the past few decades in terms of its competitive level and visibility [1]. However, this area demands further investigation, given that the literature specifically focused on female basketball [2,3], youth basketball [4], or both of them [5] remains limited.

Considering the actions involved in determining offensive performance, the main offensive actions are those performed with the ball [6]. Shooting has been identified as the most influential factor on the final result [7], demonstrating a direct correlation between shooting accuracy and the probability of victory [8]. The efficiency of shooting depends on multiple variables, the relevance of which increases in youth categories, where technical and decision-making skills are still developing [9,10].

The next most important offensive play is the pass [11], constituting a fundamental component of team play [12], but this action does not depend solely on technique but also on decision-making and perceptual ability under pressure [13], with mastery of this action reducing the main cause of ball loss in female basketball [14]. On the other hand, dribbling is linked to physical abilities such as coordination, speed, agility, quickness, and endurance [15].

In addition to ball-involving actions, collective play is also influenced by off-ball movement actions [16,17], as well as screening actions concerning both on-ball and off-ball screens [18]. However, in youth categories, the use of screening is more limited due to its complexity, with priority given to the development of basic technical and cognitive fundamentals [19,20]. This research focuses on these five offensive plays, since all of them are exclusively offensive, even though it is known that other actions, such as rebounding, play an important role in offensive performance [21,22].

Alongside technical actions, various studies have corroborated that offensive performance is conditioned by different contextual factors. Playing position is a key determinant, with specific actions registered for each position [23]. Specifically in women’s basketball, guards are involved in more dribbling and passing actions, forwards are more involved in shooting, and centers are more involved in defensive actions [2]. Likewise, ball possession time varies according to the position [24], match location shows that playing at home leads to better decision-making and a superior offensive performance [25,26,27], game period indicates that physical demands increase as matches progress, affecting technique and decision-making [28,29,30], and the zone in which the action is performed demonstrates how the effectiveness of certain offensive plays decreases and how the likelihood of errors in players’ decision-making and execution increases as proximity to the opponent’s basket increases [10,31,32]; other research indicates that these affect decision-making in other youth sports as well [33,34,35].

Therefore, the aim of the present study was to analyze the offensive participation and performance of players from a U14 female basketball team throughout a season, by evaluating decision-making and execution in offensive actions performed, according to playing position and different contextual variables.

## 2. Materials and Methods

This research was observational in nature, with a descriptive, longitudinal, and multidimensional design [36]. The recording, identification, and analysis of offensive actions and performance variables were conducted once the competition had concluded, through systematic analysis of match videos [37]. A total of 9450 offensive actions were analyzed during the 2022–2023 season across 15 regular-season matches in the U14 category (Supplementary File).

### 2.1. Participants

This research adopted a single case study design. The sample consisted of eleven players from the same team (two guards, five forwards, and four centers), with a mean age of 13.89 (±0.25) years. No players were excluded due to injury or minutes played, and none of them had previously suffered long-term injuries. All players trained for a minimum of six hours per week, divided into three two-hour training sessions. They also had prior basketball experience of three years, including at least two years of competition in regional federated leagues.

### 2.2. Observational Instrument

To ensure that each item captured the full range of possibilities for each action and each variable before the data collection and the analysis began, an ad hoc instrument was designed. Ten experts collaborated on this effort; they were provided with individual reports containing the initial definitions in an effort to avoid differences in interpretation, actions that were outside the scope of the analysis, and biases that might have arisen during data analysis by the researchers [38]. Each variable was evaluated in different terms, and the experts, through a quantitative assessment, initially agreed on unambiguity (88.5%), importance (79.8%), and relevance (83.2%). Along with the qualitative assessment they conducted, the instrument was revised, and variables that did not receive at least 60% agreement from the experts in any of the sections were eliminated. The revised version was resubmitted to the experts, resulting in unanimous agreement on the final definitions.

The dependent variables in this study were dribbling, shooting, passing, screening, and off-ball movement. For each of these, the study assessed whether the execution was effective or ineffective and whether the decision was appropriate or inappropriate (Table 1).

**Table 1:** Offensive plays analyzed, execution or decision variables, and definition.

Offensive Play		Execution/Decision	Definition
1	Dribbling	Effective	The player dribbles the ball in a controlled manner without breaking any rules.
		Ineffective	The player loses possession of the ball due to a legal steal or commits a foul while dribbling.
		Appropriate	The player has created offensive opportunities, by using the dribble, either by creating a better position on the court or by creating a better passing or shooting opportunity.
		Inappropriate	The player dribbles when she has the option to pass to a teammate in a better position or to score a basket, or she commits a foul.
2	Shooting	Effective	The player scores a basket by shooting or by finishing near the rim (layup).
		Ineffective	The player does not score, or the shot is blocked by a defender, or an offensive foul is committed during the shot.
		Appropriate	The player has a clear scoring opportunity, or the possession time or game time is about to run out.
		Inappropriate	The player takes the shot without a chance of scoring, either due to the distance of the shot or opposing defense, or because a teammate is in a better position to take it within the time remaining.
3	Passing	Effective	The pass reaches a teammate perfectly.
		Ineffective	The pass does not reach the teammate in time, or it is intercepted or touched by a defender.
		Appropriate	The pass is made to a teammate who is in a better position or has the potential to create an advantage.
		Inappropriate	The pass is made to a teammate who is not in a better position or who cannot gain an advantage.
4	Screening	Effective	The player performs the move from a static position without committing an offensive foul and creates an advantage for herself or her teammate.
		Ineffective	The player moves, commits an offensive foul, or fails to create an advantage for herself or her teammate.
		Appropriate	The screening player, or the one being screened, gains an advantage over the defenders when dribbling, passing, shooting, receiving passes, or creating open spaces on the court.
		Inappropriate	The screening player, or the one being screened worsens their attacking position prior to the screen, or a foul is committed.
5	Off-ball movement	Effective	The player’s movement has a positive effect on the spacing of the ball-handler or on team play.
		Ineffective	The player does not create space, does not hinder the defensive action, or even aids it.
		Appropriate	The player creates space to allow the ball-handler to make a move, or to allow herself to receive the ball, with less defensive pressure than initially faced.
		Inappropriate	The player makes a move that interferes with the offensive play of the player with the ball.

Five independent variables were considered, each with a different number of categories depending on the possibilities of each one, as defined in the following table (Table 2).

**Table 2:** Independent variables, degree of openness, and definition of the variables.

Variable		Degree of Openness	Definition
1	Location	Home	The team being studied is playing at home.
		Away	The team being studied is playing away.
2	Game period	First quarter	The play occurs in the first game period.
		Second quarter	The play occurs in the second game period.
		Third quarter	The play occurs in the third game period.
		Fourth quarter	The play occurs in the fourth game period.
3	Zone of action	Offensive zone	The play occurs between the offensive baseline of the studied team and the offensive three-point line.
		Intermediate zone	The play occurs between both three-point lines.
		Defensive zone	The play occurs between the defensive baseline of the studied team and the defensive three-point line.
4	Wins at the time of the matchup	More wins	The team being analyzed has more wins than its opponent at the time of the matchup.
		Same wins	The team being analyzed has the same number of wins as its opponent at the time of the matchup.
		Fewer wins	The team being analyzed has fewer wins than its opponent at the time of the matchup.
5	Player position	Guards	The player performing the action plays the guard position (2 players).
		Forwards	The player performing the action plays the forward position (5 players).
		Centers	The player performing the action plays the center position (4 players).

### 2.3. Procedure

Matches were recorded using a high-definition camera (Victure AC700 4K), (Shenzhen Apeman Innovations Technology Co., Ltd. of Shenzhen, China) positioned to capture the entirety of the court, in order to collect the data [39]. Pause and rewind tools were used to examine all actions through a double viewing, conducted by the same researcher in two different sessions (test–retest), with the aim of minimizing errors and ensuring coherence, uniformity, and objectivity throughout the analysis process, thereby contributing to the reliability and validity of the results obtained [40].

Data were analyzed using the Game Performance Assessment Instrument (GPAI), a tool that allowed for the analysis of players’ participation and game performance [41], enhanced analytical capacity by incorporating elements associated with the resolution of tactical situations in team sports [17], and differed from other performance instruments that only analyzed the execution of actions with the ball.

Furthermore, the GPAI was adapted for this research to ensure that scores ranged between zero and one [42], a solution that has been validated in other studies [43,44]. Measurements for each variable were calculated using the GPAI (Table 3).

### 2.4. Statistical Analysis

Data were processed using IBM SPSS Statistics v. 31.0 to analyze the study variables and obtain frequencies and percentages for each action.

To examine differences in the distribution of offensive actions by position (guards, forwards, and centers) and other contextual variables (match location, game period, action zone, and previous wins), the chi-square (χ^2^) test of independence was used. Adjusted standardized residuals (ASRs) were inspected to identify which categories specifically contributed to the observed associations, and the χ^2^, degrees of freedom and *p*-values were reported. Additionally, Cramer’s V coefficient was calculated as a measure of effect size. The Game Involvement Index (GI), categorical and percentage-based in nature, was similarly analyzed using χ^2^ following the same procedure. The level of statistical significance was set at *p* < 0.05 (95% confidence interval) for all tests.

Continuous variables (SEI, DMI, and GP) were analyzed using a multivariate analysis of variance (MANOVA), with playing position as the main independent factor, to examine simultaneous effects on multiple dimensions of offensive performance. Wilks’ Lambda (λ) was used as the multivariate contrast statistic, and univariate analyses of variance (ANOVA) were conducted to identify in which specific index the differences were located. Partial eta-squared (η^2^p) was calculated for effect size in all ANOVAs, and post hoc tests were applied to identify differences between positions.

Because the guard subgroup comprised only two players, the resulting within-group variance estimate for this subgroup is based on a single degree of freedom (*n* − 1 = 1), and inferential comparisons involving the guard subgroup are reported as exploratory rather than confirmatory.

## 3. Results

The overall results, based on playing position, revealed significant differences (*p* < 0.05, 95% confidence interval) for dribbling actions, in which guards were more represented compared to the other positions, and for off-ball movement actions, where forwards and centers were more represented compared to guards. The shooting action, although it did not show significant differentiation (*p* = 0.062), evidenced differences in the adjusted standardized residual between centers (8.7) and guards (−8.8) (Table 4).

The indices by position presented significant differences in game involvement (GI) (*p* < 0.001), with a higher percentage for guards (45.12%). The execution index (SEI), decision-making index (DMI), and game performance index (GP) also indicated significant differences (*p* < 0.05). The multivariate analysis of variance (MANOVA) did not reach statistical significance, but a large multivariate effect was identified (Wilks’ λ = 0.176, F (6,12) = 2.77, *p* = 0.063, η_p^2^ = 0.581), while the univariate analysis (ANOVA) revealed differences in SEI, DMI, and GP between guards and centers, and in SEI between forwards and centers, with lower indices for centers (Table 5).

Likewise, when relating the game location variable to playing position, significant differences emerged (*p* < 0.05) across all indices, with the exception of the away DMI (*p* = 0.062), with guards accounting for the highest participation rates at both venues (45.12%). Univariate analysis of variance by playing position revealed differences between guards and centers in SEI and GP when playing at home, and in SEI, DMI, and GP when playing away. Differences between forwards and centers were also observed in the visiting SEI. When dependent variables were examined simultaneously, however, the MANOVA did not reach statistical significance for home games (Wilks’ λ = 0.207, F (6,12) = 2.40, *p* = 0.093, η_p^2^ = 0.55), whereas a statistically significant multivariate effect was found for away games (Wilks’ λ = 0.114, F (6,12) = 3.91, *p* = 0.021, η_p^2^ = 0.66) (Table 6).

When analyzing the data by game periods, statistical significance was identified in the GI across variables for all periods (*p* < 0.001), as well as significant differences (*p* < 0.05) in SEI, DMI, and GP across all quarters, with the exception of the DMI in the first quarter (*p* = 0.185). Similarly, differences between guards and centers were found across all indices for all four periods, except for the DMI in the first quarter. Between forwards and centers, differences were observed only in SEI during the first and second quarters, and in GP during the first and third quarters. Differences between guards and forwards emerged in SEI during the third and fourth quarters, in DMI during the second and fourth quarters, and in GP during the second, third, and fourth quarters. Regarding the multivariate analysis, the MANOVA did not reach statistical significance in the first quarter (*p* = 0.051) but did so in the remaining periods (1st quarter: Wilks’ λ = 0.162, F (6,12) = 2.97, *p* = 0.051, η_p^2^ = 0.597; 2nd quarter: Wilks’ λ = 0.121, F (6,12) = 3.76, *p* = 0.024, η_p^2^ = 0.653; 3rd quarter: Wilks’ λ = 0.135, F (6,12) = 3.44, *p* = 0.032, η_p^2^ = 0.633; 4th quarter: Wilks’ λ = 0.211, F (4,14) = 4.12, *p* = 0.021, η_p^2^ = 0.540) (Table 7).

Concerning the court zone where actions were executed, significant differences were detected in the offensive zone for SEI, DMI, and GP, as well as in the SEI within the intermediate zone. Significant differences in the GI were also identified across all three zones, with guards recording the highest participation rate in the defensive zone (53.69%). SEI did not differ significantly between positional groups in any of the three zones. By contrast, DMI differences were found between guards and both forwards and centers, though exclusively within the offensive zone, and GP differences emerged only between guards and centers, restricted to that zone. The simultaneous evaluation of all dependent variables failed to reach statistical significance in any of the three zones examined (offensive zone: Wilks’ λ = 0.176, F (6,12) = 2.77, *p* = 0.063, η_p^2^ = 0.581; intermediate zone: Wilks’ λ = 0.338, F (6,12) = 1.44, *p* = 0.278, η_p^2^ = 0.418; defensive zone: Wilks’ λ = 0.263, F (6,12) = 1.90, *p* = 0.162, η_p^2^ = 0.487) (Table 8).

As shown in Table 9, players’ differences were examined according to the team’s wins relative to those of their opponents. Across all three win situations, guards consistently stood out, showing differences against forwards in SEI and GP, and between guards and centers in GP, when the team had accumulated more wins than the opponent. When win totals were equal, guards differed from both forwards and centers in SEI, DMI, and GP, whereas when the team had fewer wins than the opponent, significant differences were found only between guards and centers in DMI and GP. Additionally, similar patterns were identified between forwards and centers when the team had more wins than, or the same number of wins as, the opponent. The MANOVA was statistically significant only in the condition of more wins than the opponent (Wilks’ λ = 0.072, F (6,12) = 5.43, *p* = 0.006, η_p^2^ = 0.731), while failing to reach significance when win totals were equal (Wilks’ λ = 0.183, F (6,12) = 2.67, *p* = 0.069, η_p^2^ = 0.572) or when the team had fewer wins than the opponent (Wilks’ λ = 0.244, F (6,12) = 2.05, *p* = 0.137, η_p^2^ = 0.506) (Table 9).

## 4. Discussion

The aim of this research was to analyze the participation and offensive performance of the players on a female youth basketball team over the course of a competitive season, considering both execution and decision-making across the different offensive actions and their relationship with various contextual variables.

From an overall perspective, this study fully aligns with [2], which found that centers dominate the shooting efficiency, and there are no significant differences in passing performance across positions. Included, guards denoted the highest GI, as well as the highest values for SEI, DMI, and GP, followed by forwards, which is consistent with other studies, possibly due to their greater control of possession, game organization, and exposure to a higher number of decisions per offensive play [2,4,24]; however, given that only two players occupied the guard position in this team, this pattern should be interpreted as descriptive of these specific players rather than as evidence of a generalizable guard-position effect. By contrast, the lower performance of centers could be related to a lower frequency of offensive participation, given the implicit condition of performing actions close to the basket, limiting their opportunities compared to perimeter positions [2,4], in contrast to the findings reported by [23], which indicated that forwards at developmental stages performed inconsistently; this should be taken into account when designing position-specific training tasks for this specific team.

As for the technical/tactical actions analyzed, the results showed dribbling, passing, and shooting as the primary offensive actions among the players analyzed, which matches the findings of [6] for youth players of the same age range. Screening was the action that occurred least frequently during matches, consistent with prior studies in which, due to its complexity, other fundamentals were prioritized [19,20]. However, screens were performed in greater volume by centers, with this position assuming functions associated with off-ball play and the generation of advantages for teammates, in line with what has been described in other investigations [18]. In relation to off-ball movement actions, forwards and centers exhibited higher frequencies in their actions, contributing to the creation of spacing, as similarly noted in other studies [16].

With respect to game location, the results were consistent with the home-court advantage documented across youth female basketball categories, an effect shown to be smallest precisely at the U14 level and to increase with age [25]. This improvement is possibly explained by greater rest, lower perceived pressure, or familiarity with the playing environment. This age-related pattern may also help explain why the advantage did not extend uniformly across positions. The SEI of centers was slightly higher when playing away, which could be caused by a more controlled selection of offensive interventions as away players, reducing the number of actions but increasing the probability of technical success.

When considering the game period variable, guards and centers showed better execution results as the quarters progressed, in contrast to studies that have described a decline in performance as the match periods advanced [28] and, via a comparable single-team observational design, for individual technical performance [27]. Although [10] did not identify game period as a significant predictor, decision-making nevertheless did decrease across all positions as the quarters advanced, consistent with the literature indicating an effect of physical and mental fatigue on decision-making quality [28]. This pattern is probably explained by the team’s physical conditioning, which softened the impact of fatigue on technical execution [30] without preventing the progressive decline in correct decision-making—a finding that supports the need to integrate cognitive training tasks in the final stages of training sessions. An increase in offensive participation among forwards was also observed in the third quarter, presumably associated with greater rotation by the coach to balance playing loads and optimize collective performance, adapting to match demands as described in other research [29,30].

Turning to the court zone in which offensive actions were performed, differences were observed across positions, where guards, through their participation, confirmed their importance in press breaks, offensive organization, and finishing. Instead, forwards specified minimal participation in the defensive zone, while centers implied reduced participation in the mid-court zone, likely due to tactical distribution in press breaks and spatial occupation. However, offensive performance was affected across all positions; as players approached the opponents’ basket, the defensive pressure exerted by the opposing team increased, raising the probability of errors in both execution and decision-making, consistent with findings reported in previous studies [10,31,32].

Finally, this study incorporated the variable of the number of wins of both teams, revealing differences according to playing position, a variable not previously examined in the literature, although a comparable contextual factor (score margin balance) has been shown to shape individual technical performance in basketball [27]. Guards exhibited reduced participation against teams with fewer wins, and their performance improved when they had more wins than the opponent, possibly because the heightened demands of more competitive matches may have hindered their performance. Forwards, on the other hand, showed their worst performance against teams with the same number of wins, and their best performance against opponents with more wins, which could be interpreted as greater perceived pressure in balanced matches [13], contrasting with [27], in which balanced score margins were associated with the highest individual technical output. Centers, for their part, performed better when the team had more wins, possibly benefiting from a context of superiority, which, in basketball, translates into a higher volume of actions close to the basket that are simpler to execute, thereby enhancing their performance [13,23]. This corroborates [27], which states that the best technical performance for all positions occurs in specific, nonlinear competitive contexts.

The results of this study offer implications for the design of offensive training on this U14 female basketball team specifically, although it could be extended to teams with similar characteristics or ages. First, we propose deliberately increasing decision-making opportunities for centers through tasks that position them as advantage creators rather than merely finishers—for example, through one-on-one situations, on-ball screens in mid-court zones, or small-sided games in which they must read defensive help and execute actions with the ball. Second, to avoid excessive dependence on guards in possession management, it is recommended to diversify offensive initiation by implementing systems in which forwards take on ball-handling or initiate play, with the aim of redistributing the decision-making load and promoting the tactical development of all positions.

## 5. Conclusions

The results exposed the existence of differentiated offensive profiles by playing position within this U14 female basketball team, with different decisions and executions according to position and the action performed. Overall, guards revealed the highest participation rates and generated the best offensive performance, while centers had the lowest participation and performance rates.

Furthermore, differences were observed in the frequency of actions performed across positions, with guards accumulating a higher number of dribbling, passing, and off-ball movement actions than the other positions and centers also surpassing the other positions in screening actions, while all three positions performed comparably in shooting actions. Based on these findings, the need to implement position-specific training sessions for this team is confirmed for the actions most frequently performed in competitions in order to enhance performance. Accordingly, it would be beneficial for guards to work on tasks that reduce the time available for decision-making and execution, requiring rapid reading and response in dribbling, passing, and off-ball movement actions. Endurance training should also be incorporated to enhance not only physical performance but also decision-making skills. For centers, specific pick-and-roll and indirect screening tasks are recommended to increase their involvement in the game, starting with simplified options that are progressively expanded to improve their decision-making capacity.

Concerning the variables studied, a slightly superior offensive performance was observed when the team played at home, without this variable affecting the players’ individual performance. In contrast, differences by position were observed based on game period, as guards and centers increased their performance as the periods progressed, while forwards declined, leading to the conclusion that the latter should be exposed to a greater number of fatigue situations in training to address these circumstances. It was also found that proximity to the opponent’s basket, associated with greater defensive pressure from the opposing team, produced lower execution and decision-making results across all positions. Consequently, it is recommended to prioritize finishing drills in one-on-one and two-on-two situations near the basket against a progressive defense, including finishing with contact and decision-making in the paint when facing defensive help. These skills can be developed collaboratively among the three positions through small-sided games. Also, the inclusion of the number of wins accumulated by both the team and the opponent as a contextual variable revealed that competitive expectations and the perceived quality of the opponents also modulate players’ participation and offensive performance, with its influence varying across positions, thus introducing a novel variable to game analysis in basketball. All of this supports the need to design specific training sessions tailored to the individual needs and contextual challenges of each position in order to enhance both individual and collective performance.

Certain limitations must be acknowledged. The study was conducted on a single team from a specific category and context (U14 female regional basketball), limiting the generalizability of the results to other categories, competitive levels, or cultural contexts. The number of players per position was small, which may have influenced the stability of some indices and the statistical power of the analyses. Additionally, the observational nature of the study prevents the establishment of causal relationships and does not incorporate measures of internal load, perceived pressure, or tactical knowledge that would help explain the observed decision-making and execution patterns in greater depth.

In light of these limitations, future investigation should replicate this design across different youth categories, both female and male, at different competitive levels, incorporating larger samples of teams and players per position and adding defensive actions to achieve a shared understanding. It would also be pertinent to integrate psychological and physical load variables to more comprehensively analyze the relationship between context, decision-making, and offensive execution. Finally, the analysis of complete offensive sequences, rather than isolated actions, could provide valuable information about decision–execution chains and how interactions between players and specific contexts modulate the overall team performance in basketball.

## Figures and Tables

**Table 3:** Specific GPAI measures [41], adapted by [42].

GPAI Indices	GPAI Indices Calculation
Game Involvement (GI)	Appropriate decisions made + Inappropriate decisions made + Efficient skill executions + Inefficient skill executions
Decision-Making Index (DMI)	Appropriate decisions made/(Appropriate decisions made + Inappropriate decisions made)
Skill Execution Index (SEI)	Efficient skill executions/(Efficient skill executions + Inefficient skill executions)
Game Performance (GP)	(DMI + SEI)/2

Used with permission of Human Kinetics, Inc. [42], Permission conveyed through Copyright Clearance Center, Inc.

**Table 4:** Frequency, percentage, adjusted standardized residual and *p*-value and Cramer’s V coefficient by player position.

		OVERALL SAMPLE		GUARDS			FORWARDS			CENTERS			
		F	%	F	%	ASR	F	%	ASR	F	%	ASR	*p*, *V*
DRIBBLING	EFFIC	2797	36.1%	1499	41.2%	13.0	746	34.2%	−2.9	552	28.7%	−11.7	*p* < 0.001, *V* = 0.100
	INEF	318	18.6%	115	18.5%		103	19.7%		100	17.9%		
	APPRO	2664	31.7%	1473	37.5%		690	29.3%		501	23.7%		
	INAP	451	42.9%	141	41.3%		159	46.0%		151	41.4%		
SHOOTING	EFFIC	331	4.3%	129	3.5%	−8.8	90	4.1%	1.3	112	5.8%	8.7	*p* = 0.062, *V* = 0.052
	INEF	775	45.4%	273	43.8%		239	45.6%		263	47.0%		
	APPRO	947	11.3%	359	9.2%		283	12.0%		305	14.4%		
	INAP	159	15.1%	43	12.6%		46	13.3%		70	19.2%		
PASSING	EFFIC	3126	40.4%	1400	38.5%	−1.2	897	41.2%	0.2	829	43.1%	1.1	*p* = 0.673, *V* = 0.017
	INEF	383	22.5%	164	26.3%		110	21.0%		109	19.5%		
	APPRO	3200	38.1%	1441	36.7%		913	38.7%		846	39.9%		
	INAP	309	29.4%	123	36.1%		94	27.2%		92	25.2%		
OFF-BALL MOVEMENT	EFFIC	1458	18.8%	607	16.7%	−6.3	446	20.5%	3.3	405	21.0%	3.7	*p* = 0.001, *V* = 0.058
	INEF	216	12.7%	66	10.6%		72	13.7%		78	14.0%		
	APPRO	1547	18.4%	640	16.3%		471	20.0%		436	20.6%		
	INAP	127	12.1%	33	9.7%		47	13.6%		47	12.9%		
SCREENING	EFFIC	32	0.4%	6	0.2%	−4.1	0	0.0%	−6.1	26	1.4%	10.9	*p* = 0.628, *V* = 0.138
	INEF	14	0.8%	5	0.8%		0	0.0%		9	1.6%		
	APPRO	40	0.5%	10	0.3%		0	0.0%		30	1.4%		
	INAP	6	0.6%	1	0.3%		0	0.0%		5	1.4%		

EFFIC = efficient; INEF = inefficient; APPRO = appropriate; INAP = inappropriate; ASR = adjusted standardized residual; F = frequency; *p* = statistical significance; *V* = Cramer’s V coefficient.

**Table 5:** Game involvement, execution, decision-making, and game performance metrics by player position.

	Guards	Forwards	Centers	
GI (%)	45.12	28.60	26.28	χ^2^ (2) = 598.52, *p* < 0.001 **, *V* = 0.178
SEI	0.85 ^3^	0.81 ^3^	0.77 ^1,2^	F (2,8) = 11.59, *p* = 0.004 **, η_p^2^ = 0.743
DMI	0.92 ^3^	0.87	0.85 ^1^	F (2,8) = 7.11, *p* = 0.017 *, η_p^2^ = 0.640
GP	0.89 ^3^	0.84	0.81 ^1^	F (2,8) = 10.03, *p* = 0.007 **, η_p^2^ = 0.715

GI = game involvement; SEI = skill execution index; DMI = decision made index; GP = game performance. ^1^ Significant differences compared to guards; ^2^ significant differences compared to forwards; ^3^ significant differences compared to centers; * *p* < 0.05; ** *p* < 0.01.

**Table 6:** Game involvement, execution, decision-making, and game performance metrics by game location and player position.

	Home				Away			
	GI (%)	SEI	DMI	GP	GI (%)	SEI	DMI	GP
Guards	45.12	0.86 ^3^	0.93 ^3^	0.89 ^3^	45.12	0.85 ^3^	0.91	0.88 ^3^
Forwards	28.25	0.81	0.89	0.85	29.03	0.81 ^3^	0.85	0.83
Centers	26.63	0.77 ^1^	0.87 ^1^	0.82 ^1^	25.84	0.78 ^1,2^	0.84	0.81 ^1^
	χ^2^ (2) = 326.62 *p* < 0.001 ** V = 0.177	F (2,8) = 11.07 *p* = 0.005 ** η^2^ = 0.74	F (2,8) = 6.93 *p* = 0.018 * η^2^ = 0.63	F (2,8) = 9.93 *p* = 0.007 ** η^2^ = 0.71	χ^2^ (2) = 273.39 *p* < 0.001 ** V = 0.179	F (2,8) = 13.52 *p* = 0.003 ** η^2^ = 0.77	F (2,8) = 4.01 *p* = 0.062 η^2^ = 0.50	F (2,8) = 7.29 *p* = 0.016 ** η^2^ = 0.65

GI = game involvement; SEI = skill execution index; DMI = decision made index; GP = game performance. ^1^ Significant differences compared to guards; ^2^ significant differences compared to forwards; ^3^ significant differences compared to centers. * *p* < 0.05; ** *p* < 0.01.

**Table 7:** Game involvement, execution, decision-making, and game performance metrics by game period and player position.

	1st Quarter				2nd Quarter				3rd Quarter				4th Quarter			
	GI (%)	SEI	DMI	GP	GI (%)	SEI	DMI	GP	GI (%)	SEI	DMI	GP	GI (%)	SEI	DMI	GP
**G**	45.98	0.84 ^3^	0.91	0.87 ^3^	47.88	0.85 ^3^	0.93 ^2,3^	0.89 ^2,3^	42.72	0.85 ^2,3^	0.92 ^3^	0.89 ^2,3^	43.88	0.87 ^2,3^	0.93 ^2,3^	0.90 ^2,3^
**F**	26.31	0.82 ^3^	0.88	0.85 ^3^	27.13	0.82 ^3^	0.87 ^1^	0.84 ^1^	31.66	0.80 ^1^	0.88	0.84 ^1,3^	29.41	0.79 ^1^	0.87 ^1^	0.83 ^1^
**C**	27.71	0.75 ^1,2^	0.86	0.81 ^1,2^	24.99	0.78 ^1,2^	0.85 ^1^	0.82 ^1^	25.62	0.78 ^1^	0.83 ^1^	0.81 ^1,2^	26.72	0.79 ^1^	0.86 ^1^	0.83 ^1^
	χ^2^ (2) = 176.54 *p* < 0.001 ** V = 0.190	F (2,8) = 11.23 *p* = 0.005 ** η^2^ = 0.737	F (2,8) = 2.10 *p* = 0.185 η^2^ = 0.344	F (2,8) = 8.00 *p* = 0.012 * η^2^ = 0.667	χ^2^ (2) = 224.26 *p* < 0.001 ** V = 0.219	F (2,8) = 9.70 *p* = 0.007 ** η^2^ = 0.708	F (2,8) = 8.88 *p* = 0.009 ** η^2^ = 0.689	F (2,8) = 13.21 *p* = 0.003 ** η^2^ = 0.768	χ^2^ (2) = 105.21 *p* < 0.001 ** V = 0.150	F (2,8) = 15.20 *p* = 0.002 ** η^2^ = 0.792	F (2,8) = 6.79 *p* = 0.019 * η^2^ = 0.629	F (2,8) = 15.53 *p* = 0.002 ** η^2^ = 0.795	χ^2^ (2) = 116.26 *p* < 0.001 ** V = 0.157	F (2,8) = 8.85 *p* = 0.009 ** η^2^ = 0.689	F (2,8) = 7.24 *p* = 0.016 * η^2^ = 0.644	F (2,8) = 8.44 *p* = 0.011 * η^2^ = 0.678

G = guards; F = forwards; C = centers; GI = game involvement; SEI = skill execution index; DMI = decision made index; GP = game performance. ^1^ Significant differences compared to guards; ^2^ significant differences compared to forwards; ^3^ significant differences compared to centers; * *p* < 0.05; ** *p* < 0.01.

**Table 8:** Game involvement, execution, decision-making, and game performance metrics based on the zone where the action takes place and the players’ position.

	Offensive Zone				Intermediate Zone				Defensive Zone			
	GI (%)	SEI	DMI	GP	GI (%)	SEI	DMI	GP	GI (%)	SEI	DMI	GP
Guards	39.97	0.78	0.91 ^2,3^	0.84 ^3^	48.67	0.92	0.92	0.92	53.69	0.92	0.94	0.93
Forwards	30.07	0.74	0.86 ^1^	0.80	32.05	0.89	0.88	0.89	20.76	0.89	0.90	0.90
Centers	29.97	0.70	0.83 ^1^	0.77 ^1^	19.29	0.84	0.85	0.85	25.55	0.92	0.91	0.92
	χ^2^ (2) = 99.06 *p* < 0.001 ** V = 0.099	F (2,8) = 4.67 *p* = 0.045 * η^2^ = 0.538	F (2,8) = 12.23 *p* = 0.004 ** η^2^ = 0.754	F (2,8) = 7.67 *p* = 0.014 * η^2^ = 0.657	χ^2^ (2) = 316.76 *p* < 0.001 ** V = 0.255	F (2,8) = 5.02 *p* = 0.039 * η^2^ = 0.556	F (2,8) = 3.00 *p* = 0.107 η^2^ = 0.429	F (2,8) = 4.21 *p* = 0.056 η^2^ = 0.513	χ^2^ (2) = 380.84 *p* < 0.001 ** V = 0.308	F (2,8) = 1.04 *p* = 0.395 η^2^ = 0.207	F (2,8) = 1.03 *p* = 0.399 η^2^ = 0.205	F (2,8) = 1.02 *p* = 0.403 η^2^ = 0.203

GI = game involvement; SEI = skill execution index; DMI = decision made index; GP = game performance. ^1^ Significant differences compared to guards; ^2^ significant differences compared to forwards; ^3^ significant differences compared to centers; * *p* < 0.05; ** *p* < 0.01.

**Table 9:** Game involvement, execution, decision-making, and game performance metrics based on previous wins and the players’ position.

	More Wins				Same Wins				Fewer Wins			
	GI (%)	SEI	DMI	GP	GI (%)	SEI	DMI	GP	GI (%)	SEI	DMI	GP
Guards	48.10	0.87 ^2^	0.94	0.90 ^2,3^	47.00	0.85 ^2,3^	0.91 ^2,3^	0.88 ^2,3^	42.11	0.85	0.91 ^3^	0.88 ^3^
Forwards	26.85	0.80 ^1^	0.89	0.84 ^1^	26.09	0.77 ^1^	0.82 ^1^	0.80 ^1^	31.28	0.83	0.89	0.86
Centers	25.05	0.80	0.88	0.84 ^1^	26.91	0.79 ^1^	0.85 ^1^	0.82 ^1^	26.61	0.75	0.84 ^1^	0.80 ^1^
	χ^2^ (2) = 251.28 *p* < 0.001 ** V = 0.222	F (2,8) = 5.41 *p* = 0.033 * η^2^ = 0.575	F (2,8) = 5.08 *p* = 0.038 * η^2^ = 0.560	F (2,8) = 7.44 *p* = 0.015 * η^2^ = 0.650	χ^2^ (2) = 226.79 *p* < 0.001 ** V = 0.205	F (2,8) = 13.78 *p* = 0.003 ** η^2^ = 0.775	F (2,8) = 13.23 *p* = 0.003 ** η^2^ = 0.768	F (2,8) = 15.56 *p* = 0.002 ** η^2^ = 0.796	χ^2^ (2) = 159.25 *p* < 0.001 ** V = 0.138	F (2,8) = 5.56 *p* = 0.031 * η^2^ = 0.581	F (2,8) = 6.45 *p* = 0.021 * η^2^ = 0.617	F (2,8) = 6.06 *p* = 0.025 * η^2^ = 0.602

GI = game involvement; SEI = skill execution index; DMI = decision made index; GP = game performance. ^1^ Significant differences compared to guards; ^2^ significant differences compared to forwards; ^3^ significant differences compared to centers; * *p* < 0.05; ** *p* < 0.01.

## Data Availability

The original contributions presented in this study are included in the article/Supplementary Materials. Further inquiries can be directed to the corresponding author.

## Supplementary Materials

The following supporting information can be downloaded at: https://www.mdpi.com/article/10.3390/sports14090366/s1, Supplementary File: 9450 offensive actions.
